# Integrating behavioural science and epidemiology to improve early detection of zoonotic swine influenza in the Netherlands

**DOI:** 10.1016/j.onehlt.2025.101091

**Published:** 2025-05-29

**Authors:** Juliette Fraser, Ewa Pacholewicz, Peter Hobbelen, Thomas Hagenaars, Ron Bergevoet, Michel Counotte

**Affiliations:** a Wageningen Social & Economic Research, Wageningen University and Research, Wageningen, the Netherlands; b Wageningen Bioveterinary Research, Wageningen University and Research, Lelystad, the Netherlands

**Keywords:** Zoonoses, Swine influenza, Social sciences, Epidemiology, the Netherlands, Earl detection

## Abstract

**Background and objectives:**

The Netherlands faces zoonotic disease risks due to its dense human and livestock populations. The 2009 H1N1 outbreak highlighted the pandemic potential of influenza virus reassortment. Effective preparedness requires integrating behavioural and epidemiological models. Human behaviour, shaped by personal, social, and institutional factors, is critical in detecting, intervening, and treating diseases. Using the Theory of Planned Behaviour (TPB), a framework was developed integrating knowledge from the TPB to improve early detection and response, using (zoonotic) swine influenza as a case study.

**Material and methods:**

Within the framework we defined the desired outcome: timely detection and notification of symptomatic (and hypothetical zoonotic) swine influenza to prevent its spread. Actions, such as symptom recognition and disease reporting, were linked to key drivers extracted from the TPB and disease transmission modelling. Expert elicitation estimated the likelihood of action for different farmer profiles, while disease transmission modelling assessed farm-to-farm spread probabilities. Simulations integrated these probabilities to evaluate intervention effectiveness across different scenarios.

**Results:**

The framework successfully combined behavioural science and epidemiology, offering nuanced estimates of intervention effectiveness. For early detection, 95 % of farmers were estimated to notify their veterinarian within 13 days post-infection. Key factors influencing action included symptom recognition and disease spread extent. The farmer profiles influenced response likelihood, while human infections linked to outbreaks had minimal impact. Farm density and assumptions about transmission probabilities significantly affected the likelihood of spread before notification.

**Discussion and conclusion:**

The framework provides a systematic approach for integrating social and epidemiological insights to support evidence-based policies. The work can be further enhanced by complementing expert judgement with more extensive stakeholder surveys, randomized scenario presentations, and immersive methods. This pragmatic tool aids policymakers in designing targeted interventions for zoonotic disease preparedness.

## 1 Introduction

In the last decades, the Netherlands experienced outbreaks and the circulation of (zoonotic) pathogens in livestock, for example, Q-fever [1], SARS-CoV2 [2], avian influenza [[3], [4], [5]] and swine influenza [6]. The latter highlights the pandemic risk posed by the emergence of zoonotic strains, as demonstrated by the 2009 H1N1/09-influenza outbreak in humans [7]. The widespread endemic circulation of influenza A viruses in pigs, combined with the probability of reassortment with human or avian influenza, could lead to the emergence of an outbreak similar to the 2009 H1N1 outbreak, posing significant public health risks. The Netherlands, characterized by its high population density of both humans and livestock, faces a heightened risk of zoonotic disease emergence [7]. To prevent the transmission of diseases from animals to humans, it is essential to maintain vigilance and implement well-coordinated actions in response to outbreak signals. To support the policy makers in the preparedness and response phases of an outbreak, infectious disease modelling is commonly applied both in the public and veterinary health domains [8,9]. Understanding disease transmission dynamics, predicting the course of outbreaks, and predicting or evaluating the effect of interventions are important aims to inform policy and guidelines. To achieve these aims, mathematical modelling is often employed as a key tool allowing to quantify the transmission dynamics from field and/or experimental data, and subsequently extrapolate to new situations for which direct observational data is absent. In such model extrapolations, often the dynamics of the spread of the disease are simulated, using scenario assumptions for (changes in) the characteristics of the at-risk population, environment and/or intervention measures.

In addition, in preparedness for, and response to infectious disease outbreaks, human behaviour plays a crucial role. Human action is needed to detect symptoms, to implement interventions, and to seek treatment, and thus shapes the course of an outbreak. For example, individuals must seek diagnosis or treatment, or report disease presence through various means. The decision to act and the behaviours displayed are influenced by multiple drivers at different levels: personal characteristics, social environment, and the broader context encompassing value chain partners, organizations, institutions, and governmental bodies. Interventions aimed at increasing pandemic preparedness will thus almost always require the inclusion of incentives that promote desired human behaviour, including compliance with advice and regulations [10]. In the context of zoonotic diseases farmers are on the front lines of outbreaks and their actions might impact the initial diagnosis and (prevent) the spread of the disease. Understanding farmers' responses and thus key leverage points for interventions will increase pandemic preparedness and enable better anticipation of interventions' effectiveness upon outbreaks.

So far many of the outbreaks mentioned above of contagious livestock diseases have been well studied from either an epidemiological or behavioural perspective, but seldomly using integrated approaches. We currently lack approaches in which behavioural models are employed in interaction with infectious disease models in a generalizable way [11]. Such incorporation is crucial and ensures that human behaviours related to infection prevention are appropriately represented, thereby enhancing the models' accuracy and predictive power [11]. Others have highlighted the need to consider human behaviour in infectious disease modelling, and how behaviour interacts with the effect of the disease and vice versa [12]. Efforts have been undertaken, for example, to include attitude and behaviour towards vaccination in models that evaluate vaccine efficacy [13,14]. However, integration between the different models remains difficult [15]. The challenges of incorporation of behavioural aspects into epidemiological models, among other include exploration: 1) how and to what extent behaviour should be modelled, and 2) the level of detail required to model differences in behaviour [13]. For this exploration, quantitative parameters of behaviour could be identified with the help of behavioural theories that provide insights into human behaviour through frameworks that conceptualize individual decision-making [11]. For example, the Theory of Planned Behaviour (TPB) [16] offers a structured framework to understand the determinants of human actions and has been widely applied to study various behaviours of farmers [17] throughout addressing three main constructs: attitude towards the behaviour, subjective norms and perceived behavioural control [16]. Developed in the 1980s by Icek Ajzen, the Theory of Planned Behaviour (TPB) is based on the principle that intention is the primary determinant of behaviour. This intention is influenced by the three key constructs [13]. In the context of zoonotic disease prevention, the TPB can clarify how farmers' attitudes towards measures, the social pressures they experience, and their perceived control over implementing these measures influence their decision-making related to the threat of livestock infectious diseases.

Here, we explore integration of concepts from behavioural sciences (TPB) and epidemiology (disease transmission modelling). We propose a framework bridging both disciplines and providing both inventory as well as quantification of drivers that trigger early detection of zoonotic disease outbreaks based on swine influenza as a case study.

## 2 Methods

**Fig. 1:**
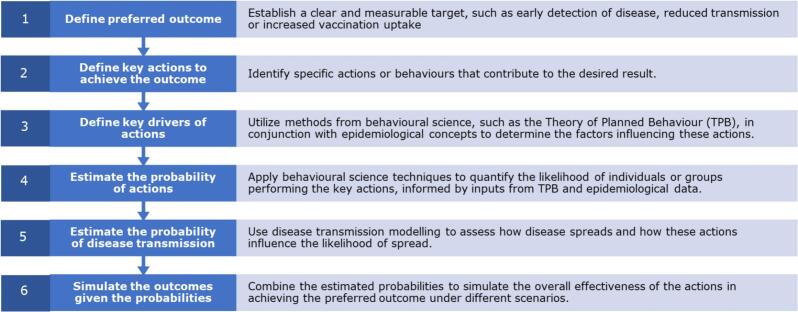
Generic approach to integrating concepts from behavioural science and infectious disease transmission modelling for outbreak and pandemic preparedness and response.

We outline a generic approach to evaluate the effectiveness of key actions in achieving desired outcomes within the scope of pandemic preparedness, where we integrate concepts from behavioural science and infectious disease transmission modelling. This methodology is grounded in challenges identified in the literature [13] and insights gathered from a collaborative workshop involving experts from both the social sciences and the fields of infectious diseases and epidemiology. The approach comprises the steps depicted in Fig. 1.

This structured framework integrates insights from behavioural science and epidemiology with quantitative modelling to systematically evaluate the potential impact of interventions on pandemic preparedness. Variability and uncertainty in the estimation of probabilities (points 4 and 5) are addressed through scenario modelling, allowing to account for the influence of different predictors and variables on these probabilities.

Throughout this work, we used ‘zoonotic swine influenza’ as a case study to illustrate concepts and to have a concrete example for stakeholders to interact with to quantify behavioural intention. Since currently circulating subtypes of swine influenza (H1N1, H3N2 and H1N2) are seldomly causing human cases [18], we thus refer here to ‘zoonotic swine influenza’ as a hypothetical strain with increased pig-human transmissibility, for the sake of providing an example that is close to reality and facilitates increasing our pandemic preparedness.

### 2.1 Definition of the preferred outcome and key actions to achieve the outcome

We defined successful ‘early detection of zoonotic swine influenza’ as the detection of a zoonotic swine influenza outbreak on a farm before it was able to transmit to other farms. Following ‘early detection surveillance ontology’ presented elsewhere [19], we consider this as the detection of a first case of a emerging disease. We further defined this as a chain of actions that needed to be taken by different stakeholders before the veterinary authority would have received the signal that ‘zoonotic swine influenza’ was present on this farm. The actions are conditional on each other, meaning that subsequent action will only be taken once a previous action was taken. Table 1 provides an overview of the actions and the stakeholders that need to take the action in other achieve the end-goal: Early detection.

**Table 1:** Overview of actions and stakeholders to achieve early detection of zoonotic swine influenza on a farm.

**Action/event**	**Stakeholder**	**Explanation**
*Start:* On farm introduction of disease	–	The moment the first infection enters the farm, marking the start of a disease outbreak
Recognition of (unusual) symptoms	Farmer/farm worker	The farmer or farm worker notices the presence of disease
Notification of symptoms to veterinarian	Farmer	The farmer notifies the veterinarian of presence of disease
Farm visit and confirmation of clinical disease	Veterinarian	The veterinarian confirms presence of clinical disease
Sampling of animals	Veterinarian/Official veterinarian	The veterinarian samples animals to achieve lab diagnosis of disease
Diagnosis of disease	Lab/Reference lab	The lab worker performs a diagnostic test on the samples and the test is positive
Notification of disease to veterinary authority	Lab/Reference lab	The lab worker reports the disease to the veterinary authority
*End:* Detection of disease	Veterinary authority	The veterinary authority takes action to contain the outbreak

To illustrate the framework's application, we focused on two primary actions of the farmer/farm worker: ‘Recognition of symptoms’, and ‘Notification of their veterinarian’. These behaviours were selected as they represent the initial and most direct steps leading to the detection of a disease outbreak on a farm.

### 2.2 Definition of key drivers of actions

The drivers of actions serve as the fundamental components of human behaviour that influence farmers' decision-making processes during a potential disease outbreak and can impact whether an action will happen or not. By identifying and categorizing these drivers, we aim to create a comprehensive model that captures the complex interplay of factors affecting farmers' behaviours.

To categorize the key drivers of the actions, we applied the TPB: Intention is the primary determinant of following a behaviour, which is influenced by the constructs of attitude, subjective norms and perceived behavioural control [16]: Attitude pertains to an individual's perception on whether a particular behaviour contributes positively or negatively to their life. Subjective norms describe the influence of social pressures manifest and the extent to which the opinion of others impact an individual's decisions. Finally, perceived behavioural control refers to an individual's sense of control over their ability to achieve goals and perform behaviours that depend on a series of interconnected actions [20].

The foundation of the theory includes external variables and background factors such as demographic details (gender, age, education, occupation, socioeconomic status, religion, etc.), personality traits (openness, conscientiousness, agreeableness, extraversion, neuroticism, etc.), and environmental influences like the physical environment and access to resources. These elements impact all other parts of the behavioural model, acting as underlying influences on the decision maker's environment [20].

To make the TPB constructs more applicable, we incorporated the TPB constructs into three distinct farmer profiles. These profiles represent typical patterns of attitudes, subjective norms and perceived behavioural control that could be observed among farmers. They provide a concrete way to understand how different farmers might respond to a disease outbreak based on their individual characteristics and perceptions. Each profile embodies a unique combination of attitudes towards farm management practices, influence of subjective norms, and perceived ability to implement control measures and adapt to challenges.

We developed the following three profiles based on literature and expert input:

1) The *‘Family-oriented farmer’* is characterized by their deep-rooted commitment to their local community and environment. These farmers exhibit a strong sense of stewardship and prioritize the stability of their farms over expansion, often with significant family involvement [[21], [22], [23], [24]].
2) The*’Business-oriented farmer’* is driven by profit and entrepreneurship. They are integrated into global market chains and focus on expanding and scaling up their operations [[21], [22], [23],^25^].
3) The ‘*Farmer without successor’* – was developed to provide a more comprehensive understanding of the diverse approaches to farming. In this profile, the farmer aims to cut costs and boost output, often sacrificing long-term viability for immediate financial gains. They are less likely to be influenced by their peers' opinions, and they feel overwhelmed by farming challenges and lack confidence in decision-making due to limited resources and support. This profile highlights the varied strategies farmers might adopt in response to contemporary challenges such as financial pressures and resource constraints, which are not fully captured by the two other profiles and allowing for a more nuanced analysis of farmer behaviour.

These profiles are stylized representations and individual farmers may exhibit characteristics of more than one profiles. Their specific traits and motivations can vary widely depending on personal circumstances, local context, and the type of farming practiced.

We hypothesized that additional and specific factors related to disease spread could influence human behaviour, primarily through their impact on risk perception. These factors include the on-farm disease progression quantified through the number of infected animals on a farm, the occurrence of human infections (potentially) associated with the outbreak, and the availability of knowledge from previous similar outbreaks.

Risk perception, while not a core element of the TPB, is assumed to shape a farmer's attitude towards disease control measures [26]. It can be defined as the subjective evaluation of a potential threat by an individual, based on the perceived likelihood of a threat occurring and the perceived impact if it does occur. In farm management, risk perception shapes a farmer's beliefs about the consequences of risk-management practices and the values attached to those consequences, thereby influencing their attitude towards implementing these practices [27]. The addition of context-specific risk perception drivers enables to explore how outbreak characteristics influence behaviour through their impact on risk perception. Table 2 gives an overview of the drivers that we further define below to estimate the probability of action. In this table, we distinguish between TPB constructs (represented by the farmer profiles) and specific risk perception drivers. This distinction allows for a more nuanced analysis of how outbreak characteristics influence behaviour, while still maintaining the theoretical framework of the TPB.

**Table 2:** Drivers that can influence the probability of action.

**Driver**	**Scenarios**	**Levels**
Attitude	Three farmer profiles	‘Family-oriented farmer’, ‘Business-oriented farmer’, ‘Farmer without successor’ (3)
Subjective norms		
Perceived behavioural control		
Risk: On-farm disease progression	Epidemic progression on the farm	Day 10, 15, 20 (3)
Risk: Human infections	Human cases linked to farm	True/false (2)
Risk: Context	Increased local threat	True/false (2)

### 2.3 Estimation of probability of action: Behavioural intention

#### 2.3.1 Survey instrument

In order to test our methodology and to estimate the probability of action (noticing symptoms and reporting disease to the veterinarian), we performed an expert elicitation. We opted for expert elicitation and not for a large stakeholder survey, since our main objective was to illustrate the conceptual framework.

#### 2.3.2 Measurement

To measure the relationship between drivers and the probability of action, we presented combinations of scenarios to stakeholders (Table 2). We developed a survey instrument in which stakeholders could provide estimates on the probability that a farmer would take action given certain circumstances. Per scenario, we provided a description of the farmers personality profile, the progression of disease, and contextual details. The progression of disease as number of pigs with typical influenza symptoms on the farm was presented as a table in which for day 10, 15 and 20 after the introduction of the infection, the total number of infected animals were provided. These were derived from a within-farm disease transmission model (described in detail below). Presence or absence of human infections related to the outbreak were displayed in the same table as a separate column. The contextual information whether an earlier outbreak of zoonotic swine influenza did occur was conveyed as well (See Supplement S2 for more details).

Stakeholders were asked to provide an estimate of a probability of the two farmer actions (recognition of symptoms and reporting these to the veterinarian) at three different time-points during a hypothetical outbreak. Each profile was presented to the expert in a concise three-paragraph format, with each paragraph linked to one of the three TPB constructs. This format illustrates whether the farmer's profile demonstrates a high or low level of the associated construct. The information was conveyed in a presentation in which profiles were represented by a pictogram and text. During the survey, the experts were presented the pictograms.

Additionally, we asked the stakeholders several validation questions in which they were asked to predict the response of the different farmer profiles. These questions were targeted to investigate the understanding of the respondents of the levels of subjective norms, attitude, and perceived behaviour control of the farmer profiles. The survey responses were analysed based on a theoretical hypothesis that each farmer profile would display distinct behavioural traits aligned with the TPB. Specifically, it was hypothesized that openness to technology adoption (attitude), influence of peers' opinion (subjective norms), and confidence in farming methods (perceived behavioural control) would vary consistently across profiles, reflecting common stereotypes or expectations associated with each type. These theoretical foundations guided the development and scoring of the validation questions to assess alignment with these anticipated behavioural patterns.

Each response to the validation question was scored on a Likert scale, ranging from ‘strongly disagree’ to ‘strongly agree’, to capture the degree of alignment with each statement. The scale enables a nuanced assessment of respondents' perceptions, with scores reflecting varying levels of agreement or disagreement. These scores provide a quantifiable means to assess the extent to which respondents perceived each profile in line with the theoretical expectations associated with subjective norms, attitudes towards innovation, and perceived behavioural control. We conducted the survey with three veterinary experts from the Netherlands in the field of swine health who work in veterinary public health institutes. See Supplement S2 for the full survey instrument.

#### 2.3.3 Analysis

We quantified the relationship between the logit of the estimated probability of action and the drivers, by assuming a linear relationship, using beta-regression [28]. We used a logit link function to map the linear predictor n_i_  to the interval (0,1). The relationship between the predictor and the variables is described in EQ. 1.

(1) $$n_{i}=\text{β0}+{\text{β1 FarmerProfile}}_{i}\;+\beta 2{\text{OutbreakDay}}_{i}+{\text{β3 HumanInvolvement}}_{i}+{\text{β4 Context}}_{i}$$

In this model:

• FarmerProfile is a categorical variable that characterizes different farmer types, influencing their likelihood of taking action based on predefined archetypes.
• OutbreakDay is a continuous variable denoting the day of the outbreak, which may reflect time-dependent factors influencing action.
• HumanInvolvement is a binary variable indicating whether human involvement in the outbreak is present.
• Context is a binary variable representing whether the context of the outbreak is favourable for action.

From the fitted beta regression model, we generated predictions for the daily probability of action using the model's parameter estimates and the observed values of the predictor variables. Predictions were computed on the scale of the response variable (probabilities between 0 and 1) by applying the inverse logit transformation to the linear predictor (n_i_). These predictions represent the expected probability of action for each day, conditioned on the specified context, human involvement, outbreak day, and farmer archetype. The resulting daily probabilities were visualized over time to highlight trends and variations in the likelihood of action, allowing us to identify patterns or influential predictors in outbreak dynamics. All computations and visualizations were performed in R (version 4.3.2) [29], and functions from the *betareg* [30,31] and *ggplot2* [32] packages.

### 2.4 Estimation of probability of transmission/spread: Disease transmission model

To estimate the daily probability of disease transmission to another farm, we developed a two-tiered model to simulate disease dynamics within and between pig farms (see Supplement S2). The within-farm model used a stochastic Susceptible-Infectious-Recovered-Maternally immune (SIRM) compartmental model implemented with the R package *SimInf* [33]. It incorporated population and disease dynamics, such as birth rates, age group transitions, and disease progression. Parameters were based on literature [[34], [35], [36]], and variability in outcomes (e.g., outbreak extinction vs. endemicity) was captured through stochastic simulations. The median infectious duration from farms with 200 sows informed the between-farm model. The within-farm model also informed the on-farm outbreak trajectory that was presented to the stakeholders to measure the probability of action.

The between-farm transmission dynamics were captured by simulations of a distance-based transmission kernel model [37], where infection probability depended on inter-farm distance and infectious duration. Using farm location data from the Netherlands, the model was parameterized via Approximate Bayesian Computation to match an end-prevalence of 40 % [38]. Sensitivity analyses tested the effects of varying key parameters. This integrated framework provided insights into transmission dynamics at both within-farm and between-farm scale.

The probability of transmission from an infected farm was considered the relevant (unfavourable) outcome and was based on the transmission kernel described above (and in Supplement S2 for details). We considered the daily probability of transmission to another farm. Since this probability depends on the density of farms around an infected farm, we randomly sampled two farms that were representative for a Dutch setting: One in a high dense area with more than 50 farms with a 5 km radius, and one in a low dense area with less than 25 farms (See supplement Fig. S5). The transmission probability to any other farm was calculated based on the fitted transmission kernel, after which the product of the complement probability was calculated. One minus this probability was interpreted as the probability that at least one other farm got infected during that day. Within these two settings we additionally considered a scenario where the daily probability of transmission was uniform over time, and one where the cumulative probability after 100 days was similar to the uniform distribution, but the daily risk was scaled to the daily outbreak size within the farm as a proportion of infectious animals. Thus, for the daily probability of between farm transmission we ended up with four scenarios: A ‘high dense uniform’, ‘high dense proportional (to outbreak)’, ‘low dense uniform’ and ‘low dense proportional’ scenario.

### 2.5 Competing probabilities: Between farm transmission versus detection

To assess whether there was disease detection before spread to other farms per scenario, we considered two competing probabilities: The probability that the infection spreads to another farm (p_spread_) and the probability of detection (p_detection_). The latter is the result of p_action2_ (probability of notification of the veterinarian) which is conditional on p_action1_ (probability of noticing symptoms) or p_detection_ = P(Action1) * P(Action2|Action1). We thus assumed that successful detection was achieved when both actions were taken. For each combination of scenarios, we ran 1000 simulations in which we sampled for 100 days from the daily probabilities, where we assumed a Bernoulli trial with a probability p. The first day for which we sampled a ‘success’ was considered that the day the event (detection or spread) took place. We then compared the day at which spread occurred with the day that the detection occurred. When spread occurred before detection, we considered this an ‘outbreak’. We report the percentage of simulations that resulted in outbreaks per scenario. In total we compared 48 scenarios (Table 3).

**Table 3:** Overview of the scenarios for the probability of detection (p_detection_) and the probability of between farm transmission (p_spread_).

**Outcome**	**Variable**	**Levels (n)**
P_detection	Farmer profile	Three farmer archetypes (3)
P_detection	Human involvement	Yes/no (2)
P_detection	Context	No information/Outbreak across the border (2)
P_spread	Farm density	Low/high (2)
P_spread	Daily risk profile	Uniform/proportional to on farm infections (2)

## 3 Results

### 3.1 Probability of action

The control questions from the survey showed overall consistency, with participants largely aligning on the ‘Family-oriented farmer's’ external influence, resistance to adopting new technologies, and confidence in established methods. However, divergences were noted in perceptions of subjective norms for the ‘Business oriented farmer’ and the ‘Farmer without successor’ profiles, likely due to differing interpretations of the influence by others. The full interpretation of the control questions is provided in the Supplement S3.

**Fig. 2:**
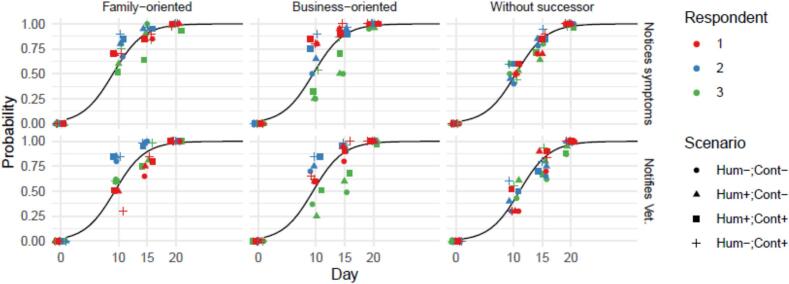
Probability of action as function of time in the outbreak. The smooth lines show the predicted probability from the fitted beta-regression model, the points show the response for each scenario, and time-point, stratified by respondent (colour) and scenario (shape). Panels show the farmer profile (columns) and action of interest (rows). Abbreviations: Hum: Human involvement in the outbreak: Yes (+) or no (−), Cont: Contextual information about earlier outbreaks known (+) or unknown (−).

We found that the estimated median probability of noticing symptoms on day 10 of the outbreak was 0.6 (range: 0.25–0.9), increasing to 1 (range: 0.93–1) by day 20. The estimated median probability of reporting disease to the veterinarian was 0.6 (range: 0.25–0.85) on day 10 and 1 on day 20 (range: 0.87–1). Part of the variability between the estimates within the same day was caused by the different scenarios, and the different farmer profiles. However, between-respondent variability was present as well (Fig. 2).

The beta-regression showed a strong relation between the extent of the outbreak and the probability of both actions: For each day later in the outbreak, the expected log-odds increased by 0.38 for noticing symptoms, and for notifying the veterinarian (Table 4). Similarly, the farmer profile ‘No successor’ showed a lower likelihood for both actions. Human involvement and context were judged to be drivers of the probability of actions for some respondents (Supplement 3, individual results), but overall they did not significantly change the probability of action. From this followed a median time to notification of the veterinarian after an on-farm introduction of an infection of 8 days (95 % CI: 4–12 days) for the ‘Family-oriented farmer’, 9 days (95 % CI: 5–12) for the ‘Business-oriented farmer’, and 10 days (95 % CI: 6–13) for the ‘Farmer without successor’.

**Table 4:** Effects of farmer profile, time in the outbreak (Day), human involvement and context knowledge on the probability of noticing symptoms and the probability of notifying the veterinarian as obtained by beta-regression.

Variable	Estimate	Std. Error	*Z*-value	*P*-value
**Outcome: Noticing symptoms**				
(Intercept)⁎	−3.467	0.182	−19.093	0
Farmer: Business-oriented	−0.179	0.173	−1.033	0.301
Farmer: Without successor	−0.520	0.180	−2.895	0.004
Day	0.383	0.016	23.772	0
Human: TRUE	−0.193	0.142	−1.362	0.173
Context: TRUE	0.185	0.143	1.299	0.194

**Outcome: Notifying the veterinarian**				
(Intercept)⁎	−3.692	0.217	−17.021	0
Farmer: Business-oriented	−0.062	0.168	−0.368	0.713
Farmer: Without successor	−0.658	0.177	−3.717	0.0002
Day	0.386	0.016	24.643	0
Human: TRUE	−0.081	0.140	−0.580	0.562
Context: TRUE	0.235	0.141	1.670	0.095

⁎ The profile for the ‘Family-oriented farmer’ served as reference category.

### 3.2 Probability of between farm transmission

**Fig. 3:**
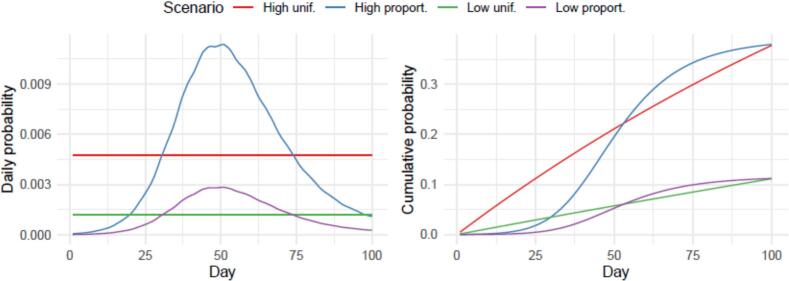
Probability of farm-to-farm transmission over time. The daily (A) or cumulative (B) probability that an infected farm infects at least one other farm over time. The scenarios are based on the farm-density (high vs low) and assumed distribution of the risk over time (uniform [unif.], or proportional [proport.] to the number of infectious animals on the farm).

We found that the daily probability of transmission to at least one other farm in a high-dense area was approximately a 4-fold higher than in a low-dense area (4.7e-3 vs 1.2e-3, Fig. 3). Given these probabilities, 5 % of the farms in a similar setting would have caused an infection in another farm after 11–49 days, depending on the scenario (Table 5).

**Table 5:** The number of days after the introduction of the infection on the farm for which 1, 5, or 10 % of the cumulative risk of spill-over to at least one other farm was reached by scenario based on the farm-density (high vs low) and assumed distribution of the risk over time (uniform, or proportional to the number of infectious animals on the farm).

**Scenario**	**Days at 1 % risk**	**Days at 5 % risk**	**Days at 10 % risk**
High uniform	2	11	22
High proportional	21	33	40
Low uniform	9	43	89
Low proportional	31	49	76

### 3.3 Conditions under which successful early detection was achieved

**Fig. 4:**
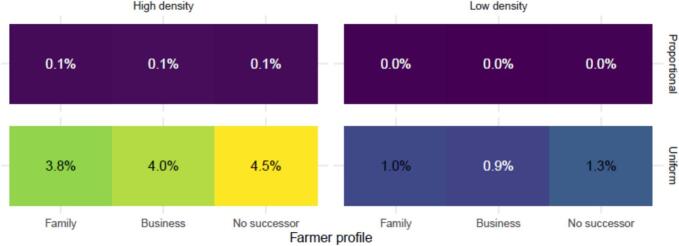
Proportion of outbreaks that spread to other farms before detection stratified by Farmer profile. Facets: Between farm risk (columns: high density vs low density areas; rows: proportional with number of infectious animals, or uniformly distributed risk). X-axis: Farmer profile (Family oriented [Family], business-oriented [Business], without successor [No successor]).

We found that the proportion of on-farm outbreaks of zoonotic swine influenza that resulted in spread to other farms before the veterinarian was notified, was the highest in the pig-high density areas, where the risk was assumed to be uniformly distributed (Fig. 4). There 3.8–4.5 % of the on-farm outbreaks resulted in transmission to at least one other farm before the veterinarian was notified. The behavioural drivers associated with the different farmer profiles did result in small differences in outbreak risk. The profile of the ‘Farmer without successor’ returned the highest outbreak risk.

## 4 Discussion

Here we have shown that we can successfully integrate concepts from behavioural science, disease transmission modelling and epidemiology to create more nuanced estimates of the effectiveness of interventions in the pandemic preparedness domain. We described a method that allows to first estimate probabilities of transmission and the required human actions that would counter transmission. We then sampled from these competing probabilities to arrive at a single metric of the probability of an outbreak. For the use-case ‘early detection of zoonotic swine influenza’, we found that it was estimated that 95 % of the farmers would notify their veterinarian within 12–13 days after an infection was introduced on a farm, with little difference between the farmer profiles. Both the probability that farmers notice symptoms and report disease to the veterinarian were driven by the extent of spread, expressed as the number of days after the infection was introduced on the farm. The perceived speed of farmers' actions showed little variation based on their profiles. Farmers without a successor were slightly less likely to act quickly, but the uncertainty intervals overlapped across all farmer profiles Human infections linked to the outbreaks were not significantly altering the proportion that took action. Farm density, but mainly the assumption on the shape of the distribution of the probability of transmission to another farm, drove the proportion of outbreaks that spread to one or more farms before the veterinarian was notified.

The results of the case study indicate that increasing the understanding of the transmission and response in high risk areas is crucial to be able to perform targeted (risk-based) interventions, which is in line with earlier findings [39,40]. Predicting how people respond to interventions in these settings, based on theories embedded in the social sciences will help identify weak spots, and will result in an even finer scale of targeting of interventions. Communication style and the implementation of the interventions can be aligned with the needs of the target groups [17]. Although we found no marked differences in actionability between the different farmer profiles, the effect of interventions might still differ based on their characteristics and personality traits.

A main strength of the framework that we present here, is that it follows a generic line of thought, making it applicable to any setting where human behaviour is required to counter disease transmission. Through-out the field of outbreak and pandemic preparedness, many of the interventions require people to take certain actions, whether it is people opting to vaccinate, notify or test for disease, or adjusting their behaviour to diminish disease transmission risk. With the framework we make each step explicit and thus transparent, helping policymakers come to decisions based on best available evidence. For researchers and practitioners, it helps identify the sources of greatest uncertainty, and thus where research or additional data collection is needed.

Our work has several limitations as well. What we present here as a probability of action, is a behavioural intention as judged by experts. The disconnect between intention and actual behaviour is a well described phenomenon [41]. However, in a hypothetical setting of preparedness for an expression of a disease that has not been encountered yet, it is hard to quantify this gap, simply because the behaviour has not been observed yet. Historic data on the outbreak response of earlier outbreaks might offer some insights, however differences in context will always remain.

Choosing to work with farmer profiles was well-adapted to the study and available resources; however, this approach presents several limitations to be acknowledged. Firstly, these profiles can oversimplify the diversity of behaviours and decision-making processes among farmers, potentially leading to generalized conclusions that do not reflect individual or contextual circumstances. The profiles used in this study were specifically developed for the Dutch pig farming context, which is characterized by a high degree of consolidation, with the number of registered pig farms decreasing from 12,894 in 2000 to 3186 in 2023, while the average herd size increased from 903 to 3398 over the same period [36]. As such, smallholders were not represented in the profiles. Similarly, organic pig farms were not explicitly considered, as they comprised only 6 % of all Dutch pig farms in 2023 [42]. However, organic and small-scale farms can be encompassed within the family-oriented farm profile. These profiling choices highlight the need for careful contextualization when applying the framework to different settings. Another limitation of the profiles is that they do not account for cultural and regional variability, which are critical factors influencing farming practices. To address these limitations in future studies, qualitative research methods such as interviews or focus groups could be employed to gather deeper insights into the motivations and experiences of various farmer groups. Lastly, we observed that the interpretation of the profiles by the experts, as evaluated by control questions, deviated between them.

To tackle the limitation of the indirectness caused by relying on expert judgement, we can replace this by a well-designed survey, where stakeholders are targeted directly. There we could directly measure the behavioural drivers [[43], [44], [45]] and their response to the different outbreak scenarios. Assuming that one would be able to recruit a sufficient sample size, stakeholders can be asked on which day they judge that they will take a certain action. The resulting ‘time-to-action’ data that is thus obtained can then be analysed using survival analysis. We must be aware that there remains a risk of anchoring when performing expert/stakeholder elicitation [46], where the order of the scenarios that are presented to the stakeholder might thus influence the results. Randomization of the order in which scenarios are presented can address this. Exploring how more immersive methods, such as serious gaming [47] or virtual reality [48], of presenting outbreak scenarios could trigger a more ‘life-like’ response would be a logical extension of the work. Similarly, it would to worth exploring when the involvement of human infections do trigger an increased willingness to report, since we did expect outbreaks that affected humans as well to increase the actionability of the farmers. It might be that scenario's where the severity is increased, or the way the information is conveyed does indeed trigger that response.

Here, we presented and tested a framework for integrating concepts from behavioural sciences and epidemiology in which we can consider the full chain of actions required to achieve a desired outcome. We have arrived at a level of detail of model integration which is pragmatic and applicable, as illustrated by the use-case. Applying this methodology will support decision-makers in their efforts to develop evidence-based policy for outbreak and pandemic preparedness in which they can consider evidence from both social sciences and epidemiology.

## Funding

This project was funded by Wageningen University and Research (ERRAZE@WUR - Early Recognition and Rapid Action in Zoonotic Emergencies) and the Dutch Ministry of Agriculture, Fisheries, Food Security and Nature (BO-43-111-102.03).

## CRediT authorship contribution statement

**Juliette Fraser:** Writing – review & editing, Writing – original draft, Validation, Methodology. **Ewa Pacholewicz:** Writing – review & editing, Validation, Project administration. **Peter Hobbelen:** Writing – review & editing, Methodology, Investigation, Formal analysis, Conceptualization. **Thomas Hagenaars:** Writing – review & editing, Validation, Methodology. **Ron Bergevoet:** Writing – review & editing, Supervision, Methodology, Investigation, Conceptualization. **Michel Counotte:** Writing – original draft, Visualization, Validation, Supervision, Project administration, Methodology, Investigation, Funding acquisition, Formal analysis, Conceptualization.

## Declaration of competing interest

The authors declare the following financial interests/personal relationships which may be considered as potential competing interests:

Michel Jacques Counotte reports financial support was provided by Dutch ministry of agriculture fisheries food security and nature. Michel Jacques Counotte reports financial support was provided by Wageningen University & Research. If there are other authors, they declare that they have no known competing financial interests or personal relationships that could have appeared to influence the work reported in this paper.

## Data Availability

Data will be made available on request.

## Appendix A Supplementary data

Supplementary material Image 1
