# Supplementary material for: Integrating behavioural science and epidemiology to improve early detection of zoonotic swine influenza in the Netherlands
Source: One Health. 2025 May 29;20:101091. doi: 10.1016/j.onehlt.2025.101091 (PMC12167494; doi:10.1016/j.onehlt.2025.101091)
Supplement: Supplementary file 1 — Supplementary material [file mmc1.docx]

Supplementary material

- S1. Survey
- S2. Model description
- S3. Detailed results/interpretation

## S1. Survey

### Introduction survey

To better understand and predict how a disease outbreak is detected early, understanding human behaviour is essential. We (researchers from Wageningen University and Research) are working on a methodology to measure behavioural intention (the intention to act) and link it to disease spread models. This way, we can better prepare for outbreaks of (animal) diseases.

The purpose of this questionnaire is to test the methodology to estimate the relationship between stakeholders' willingness to act under different circumstances. In this questionnaire, we present various scenarios. We describe a type of personality (character), a type of outbreak, and contextual information.

We ask you to estimate the willingness to act of a character who is experiencing a certain disease outbreak. We ask you to put yourself in this persona's shoes and, using the provided information, make an estimate at three different points in time during the outbreak. We are interested in whether you think it is likely that the character will recognize the disease (action 1) and subsequently report the disease to their veterinarian (action 2).

The described persona and scenarios are hypothetical and are solely for testing the methodology. The results will be analysed anonymously, with the respondent being identified only by a random, non-traceable identification number.

The survey consists of four parts: 1) this introduction, 2) the introduction of the persona, the description of the disease outbreak and the context, 3) control questions about the persona, 4) four different scenarios.

### Introduction persona, disease outbreak and context

**Persona (Farmer profiles):**

**
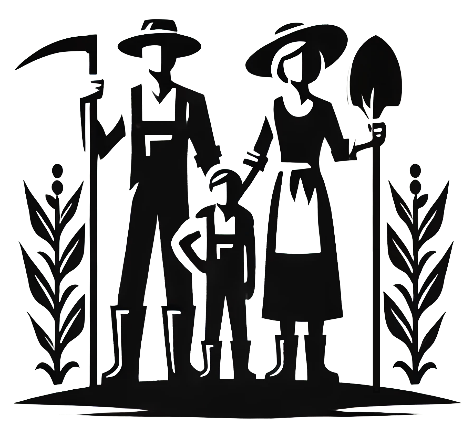
**

**A:** The **family-oriented farmer** starts work at the pig farm before dawn. He is committed to **preserving agricultural heritage** through technical skills and **generational knowledge**, while prioritizing **environmentally sustainable practices.** The large farm, with **significant sales**, is a family-driven operation focused on traditional farming, avoiding diversification and activities like agritourism.

Fellow farmers, neighbours, and family members’ opinions play a **significant role** in shaping their practices and decisions.

They feel **confident** managing the farm efficiently using **proven techniques**, which gives them a **sense of control**.

**
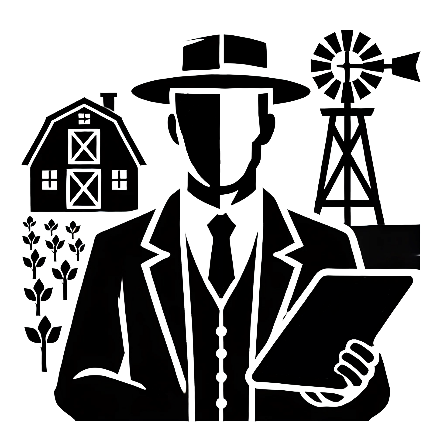
**

**B:** The **business-oriented farmer** focuses on **optimizing production and maximizing profits**. Running a large, efficient farm connected to global markets, they invest in modern housing, automation and equipment. External labour, both domestic and from EU member states, is crucial for operations.

**Market demands, industry trends,** and **financial stakeholders** shape their decisions. They maintain **strong networks** with other successful farmers and agribusiness experts, relying on industry and financial advice for their strategies.

This farmer focuses on **maximizing output** and **cutting costs** through **advanced technology** and **data-driven strategies**. Confident in managing the farm with **high-efficiency systems**, they view external labour as a key input factor and are less concerned about obstacles like **getting bank loans approved**, or **encountering issues with insufficient knowledge and available resources**.


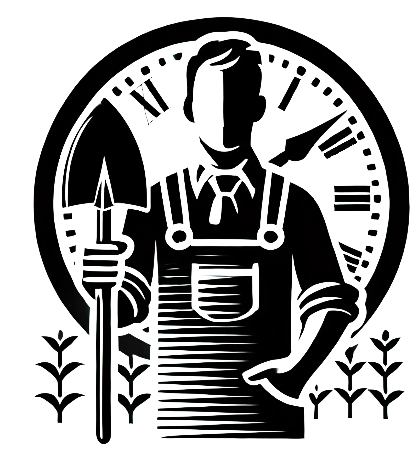


**C: The farmer without successor** focuses on **avoiding expenditures**, prioritizing **immediate** **profit** **over** **long**-**term** **sustainability** or ethical concerns. With **no** **foreseen** **successor**, their actions are driven by practical, **short**-**term** **goals**.

This farmer is influenced by **others** **who** **also** **value** **efficiency** and financial results. He is **less** **concerned** with the **views** **of those who emphasize sustainability** or **ethical** **practices**.

The farmer feels **overwhelmed** by **farming's** **challenges** and **uncertainties**, lacking confidence in making decisions due to **limited** **resources** and **weak** **support** **networks**. This mindset leads to **hesitation** in **adopting** **new** **practices** or **technologies**.

---

**Disease outbreak:** The pig farmer works on a closed pig farm. It is a farm with 300 sows. At some point, with the purchase of new gilts, a disease is introduced to the farm. Increasingly more animals become sick: they have fever, they cough, and their appetite is reduced. It starts with a few animals coughing, but soon a larger outbreak develops (see table below).

| **Days after introduction of disease** | **Number of diseased pigs** | **Number of diseased humans** |
| --- | --- | --- |
| Day 5 | 5 | 0 |
| Day 10 | **15** | **1** |
| Day 15 | **40** | **3** |
| Day 20 | **100** | **4** |

**Human involvement in the outbreak:** We consider two possibilities:

- **A:** Only pigs get sick.
- **B:** As time progresses, people also become sick, initially an employee, but later also people with whom the employee has been in contact. The pig farmer is informed about this (see table above, column ‘ Number of diseased humans’).

**Context:** The pig farmer is aware of the current situation regarding animal diseases. We consider two possibilities:

- **A:** There is a 'normal' threat of animal disease outbreaks, there are no concerning outbreaks near the pig farm.
- **B:** There is an increased threat: Just across the border, there has been an outbreak of a 'flu-like' swine disease that has also affected humans.

**Outcome:** We are interested in two actions necessary to detect the disease outbreak: 1)The pig farmer notices that there is a disease of interest is spreading that may need to be reported ('notices'). 2) The pig farmer contacts his or her veterinarian ('reports to vet').

We ask you to answer how likely you think it is that the pig farmer will notice the disease and subsequently reports the disease to their veterinarian. We use a scale to express the probability:

In part 3 of the survey, we ask some control questions about the persona. In part 4, we present a different combination of persona, outbreak, and context each time and ask you to estimate the likelihood of the outcome.

### Control questions

You will be presented with three questions to assess your understanding of the farmer profile.

[Control question relating to attitude]: Please rate your agreement with the following statement: “The farmer uses new technologies”:

- Strongly disagree
- Disagree
- Somewhat disagree
- Neutral
- Somewhat agree
- Agree
- Strongly agree

[Control question relating to subjective norms] Please rate your agreement with the following statement: “The farmer’s decisions about farm management are influenced by the opinions and expectations of others”:

- Strongly disagree
- Disagree
- Somewhat disagree
- Neutral
- Somewhat agree
- Agree
- Strongly agree

[Control question related to Relates to perceived behavioural control] Please rate your agreement with the following statement: “The farmer has high confidence in their farming methods”:

- Strongly disagree
- Disagree
- Somewhat disagree
- Neutral
- Somewhat agree
- Agree
- Strongly agree


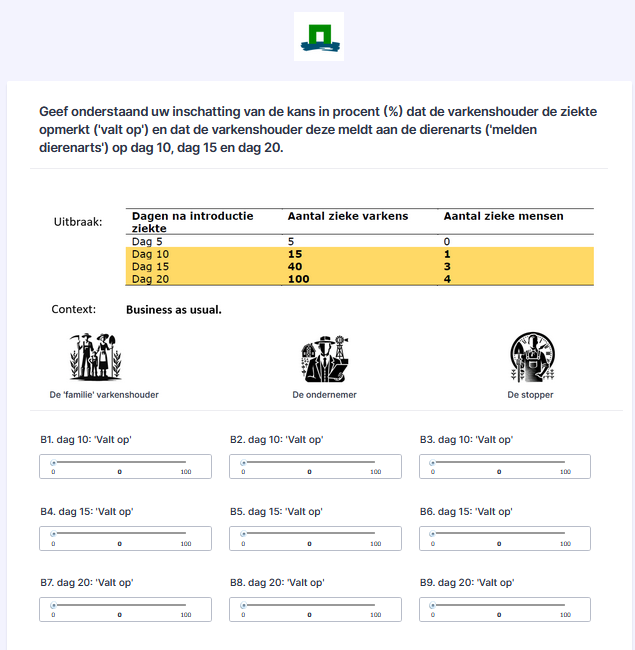


**Figure S1.1**. Screenshot of the online survey (in Dutch) in which experts were asked to provide their judgement of the probability of actions using a slider for each time point and farmer profile.

## S2. Disease transmission model

Infectious disease dynamics within a pig farm are governed by different processes. Both the disease properties and the farm management influence the natural course of a disease outbreak. Farm management, for example, drives the intensity of contact between animals and the frequency with which new susceptible individuals are added. Within the farm, we modelled the disease dynamics using a stochastic compartmental model. Between pigs farms we considered that the disease dynamics could be described using a transmission kernel [1]. We constructed both a ‘within farm model’ and a ‘between farm model’, the infectious duration, the time that at least one infectious animal was present on the farm, was used to fit the between farm model; the between farm model informed the probability of transmission to another farm at farm level (Figure S2.1).


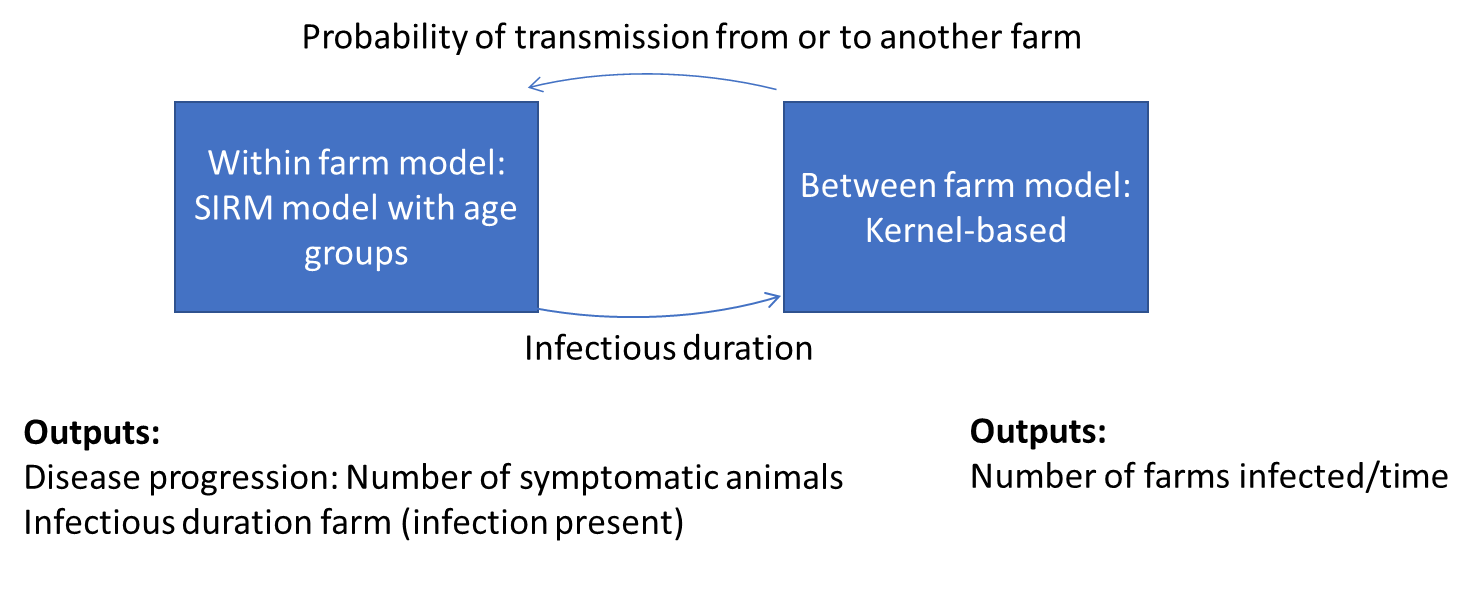


**Figure S2.1.** The interaction between the ‘within farm model’ and the ‘between farm model’. Output from the within farm model (the infectious duration) is used to parameterise the between farm model. Subsequently the between farm model informs the ‘probability of transmission to another farm’ at the farm level.

### Within farm model

We use a compartmental model in which dynamics between different disease states are expressed by ordinary differential equations. Here, animals can exist as susceptible, infectious, recovered or maternally immune individuals (SIRM), similar to [2, 3]. Population dynamics, birth, ageing, and moving out of the farm are implemented as ’post-time-step events’ as defined in the R package *SimInf* [4]. See Figure S2.2 for a graphical depiction of the model. In the baseline model, we assume homogeneous mixing between groups and transmission (β) and recovery (γ) rates to be the same for all groups. In order to calculate the disease incidence (or new cases per age group), we introduce a dummy compartment (C, not depicted in the figure) which represent the cumulative incidence.

Population dynamics were implemented as *SimInf* ‘events’, in which groups of animals move to a subsequent age-group at discrete timepoints. We assumed 2.4 litters per sow per year, with an average litter size of 14 piglets. Piglets were weaned after 30 days, and after another 40 days they moved to the fattening group. Sows were replaced with susceptible new sows with a rate of 0.4/year (table S2.1).


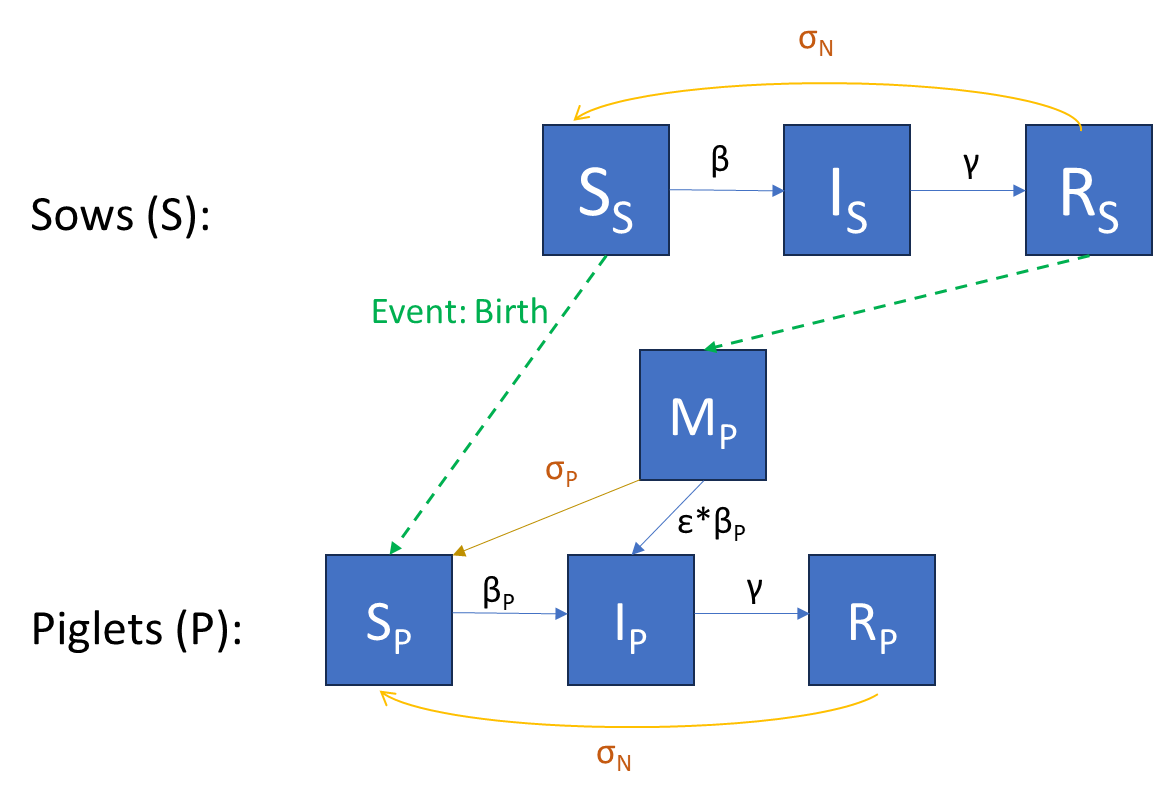


**Figure S2.2.** Graphical representation of the Susceptible-Infectious-Recovered-Maternal immune (SIRM) compartmental model in which animals exist in age-classes sow (S), piglet (P), weaned piglet (W, not shown here) and fattening pig (F, not shown here). Piglets born from immune sows (R_S_) have maternal antibodies (M_P_) that wane over a period of 11 weeks (σ_P_=1/77 days^-1^).

Parametrisation of the model was based on literature existing literature (Table S2.1). Disease progression was modelled based on the number of incident cases (C). After a latent period of 2 days, animals remained symptomatic for 5 days. We allowed 175d as burn-in period, to populate the farm based on an initial number of sows. After the burn-in period, 5 infectious sows were introduced. Figure S2.3 gives an example of the median number of symptomatic animals from the day of introduction for a closed farm of 200 sows.

**Table S2.1.** Parameters used to describe the disease dynamics and the population dynamics of the within farm model.

| Parameter | Value | Description | Source |
| --- | --- | --- | --- |
| R0 | 1.5 | β/γ | Calculated |
| β_n_ | 0.225 days-1 | Transmission rate for age group n | [2] |
| γ_n_ | 0.15 days-1 | Recovery rate for age group n | [2] |
| σ_p_ | 1/77 days-1 | Loss of (maternal) immunity (piglets) | [2] |
| σ_N_ | 1/180 days -1 | Loss of immunity acquired after infections | [2] |
| Introduction size | 5 | Five infected sows are introduced | Assumed |
| Introduction time | 175 days | After a ’burn in’ period when farm is fully populated | Assumed |
| p | 1 | proportion symptomatic | Assumed |
| Latent period | 2 days | days until onset of symptoms | [5] |
| Symptomatic period | 5 days | duration of symptomatic period | [5] |
| Litter interval | 7 | Days between litters | [6] |
| Litter size | 14 | Number of piglets per litter | [6] |
| Litters/year | 2.4 | Number of litters per year per sow | [6] |
| Replacement rate sows | 0.4 | Sows are replaced after 1/0.4=2.5 litters | [6] |
| pd | 30 | Days as ’Piglet’ (farrowing) | [6] |
| wd | 40 | Days as ’Weaning’ | [6] |
| fd | 100* | Days as ’Fattening’ | [6] |


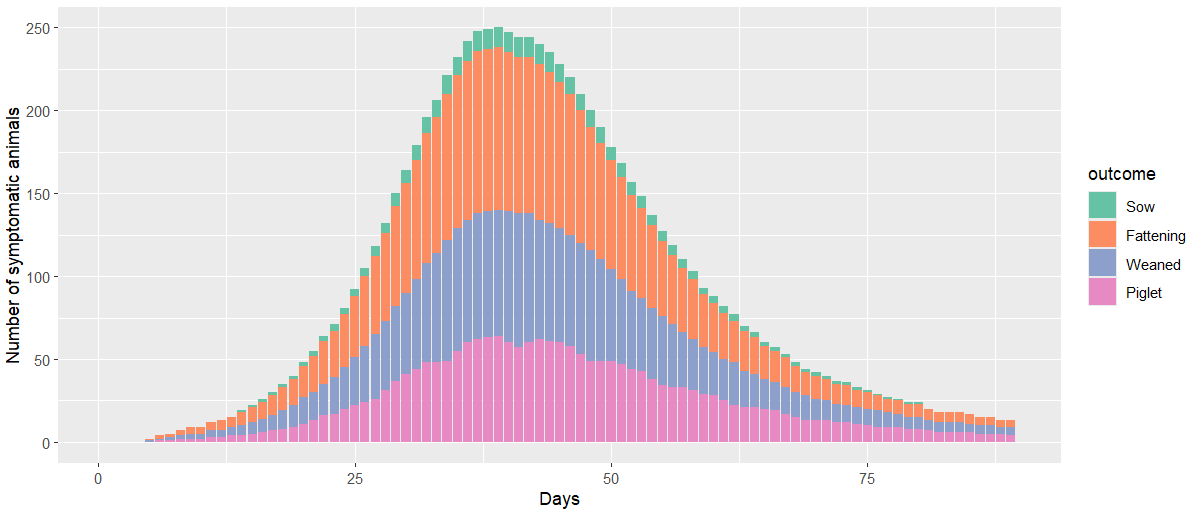


**Figure S2.3**. The median number of symptomatic animals per age group over time in a farm with 200 sows.

Due to the stochastic nature of the disease, we observed that a proportion of the introductions resulted in outbreaks that died out (Figure S2.4), and a proportion in endemic circulation within a farm, where the virus continued to circulate due to population renewal and loss of (maternal) immunity. These proportions were depended on the farm size, in line with earlier observations [3]. The simulated infectious duration of a farm with 200 sows, or a total farm size of 2795 pigs, given a closed farm, was used to estimate the parameters for the between farm model.


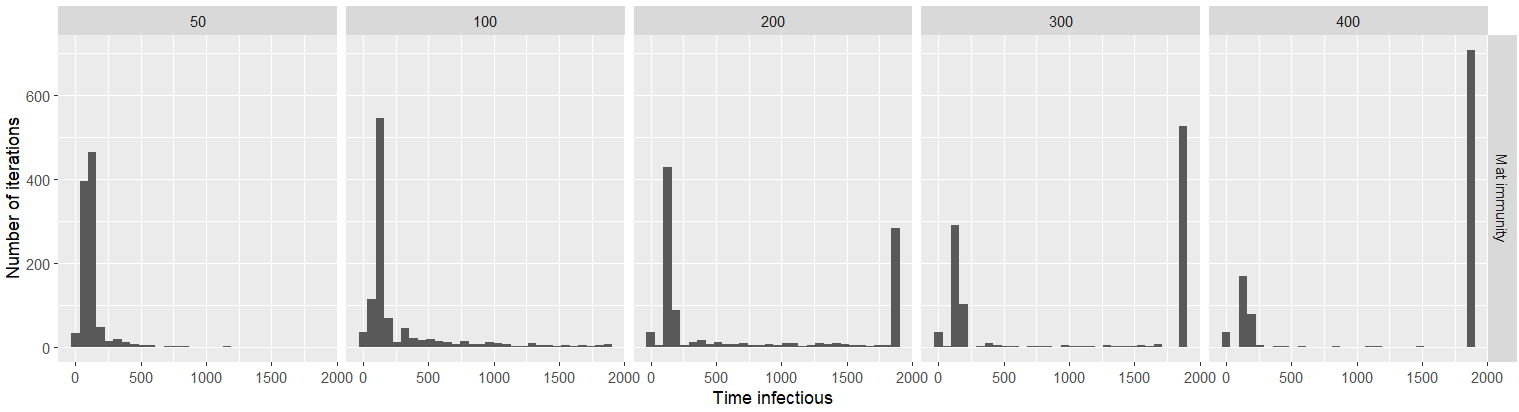


**Figure S2.4**. The duration of an outbreak (Time infectious) for 1000 iterations per scenario with number of sows per farm (50, 100, 200, 300, 400, as column facets).

### Between farm model

To model the transmission between farms, we applied a kernel based approach [1]. Transmission is assumed to decrease with the distance between farms and the transmission kernel describes the daily transmission hazard (*h*) between an infectious and susceptible farm as a function of $r_{ij}$, the Euclidean distance between an infectious (*i*) and a susceptible farm (j), r0 is the ‘kernel offset’, h0 is the amplitude of the transmission kernel indicating the transmission hazard for very small distance and $\alpha$ is a scaling exponent (EQ S2.1).

$h\left( r_{ij} \right)=\frac{h0}{(1+{r_{ij}/r0)}^{\alpha}}$ (EQ S2.1)

The probability that an infected farm infects a susceptible farm is a result of the infection hazard (h) and the infectious duration of the infected farm (T*_i_*) (EQ S2.2) [7].

$p_{ij}=1-e^{-h\left( r_{ij} \right)T_{i}}$ (EQ S2.2)

From that follows that the probability that a single infected farm will infect at least one other farm within a certain time (T*_i_*) is given in Equation S2.3, or one minus the probability that all farms escape infection.

$1-\prod_{j=1}^{n} ({1-p}_{ij})$ (EQ S2.3)

To parameterize the model, the kernel was implemented in a stochastic framework in which we considered multiple stochastic events: Transmission or escape from infection, and in case of infection: recovery or escape from recovery. In case of infection, the infectious duration was sampled from distribution of the infectious duration of the within farm model for a farm of 200 sows.

We used data location data of pig holdings in the Netherlands on which more than 100 pigs were present (Figure S2.5). We assumed (1) no distinction between farm types, (2) no cross immunity for the emerging zoonotic strain, (3) no within-host competition between emerging and existing strains, and (4) infected farms are immediately infectious (no latent period at farm level).


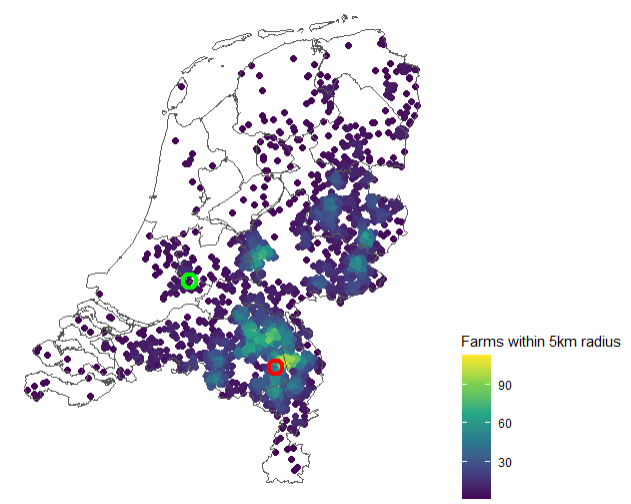


**Figure S2.5.** Distribution of pig farms within the Netherlands with more than 100 animals/holding (point) and the number of farms within a 5 km radius (colour), selected farms that represent a high-dense (red circle) and a low-dense (green circle) area.

The kernel was fitted to an end prevalence where 40% of the Dutch farms experienced infection [8], with an assumed r0 (the ‘kernel offset’) of 1, and a scaling exponent ($\alpha$) of 1.6, based on earlier experience [1]. Infection was seeded in a low pig-dense area and a high pig-dense area (Figure S2.5). An Approximate Bayesian Computation (ABC) approach was used to parameterize the kernel amplitude (*h*0). We applied an ABC-SMC algorithm described by Toni et al. (2009) [9]. We evaluated the effect of the kernel offset (default of r0=1) on the proportion of introductions that lead to endemic circulation and the end-prevalence given endemic circulation, by increasing and decreasing the value to 1.5 (higher r0) and 0.5 (lower r0) (Table S2.2, Figure S2.6). The values were robust against these changes?.

**Table S2.2.** Parameter estimates for the kernel amplitude (h0).

| **Scenario** | **2.5%** | **25%** | **median** | **mean** | **75%** | **97.5%** |
| --- | --- | --- | --- | --- | --- | --- |
| Lower r_0_ | 0.000392 | 0.000417 | 0.000435 | 0.000437 | 0.000459 | 0.000483 |
| Default r_0_ | 0.000153 | 0.000162 | 0.000167 | 0.000167 | 0.000172 | 0.000181 |
| Higher r_0_ | 8.73E-05 | 9.31E-05 | 9.64E-05 | 9.67E-05 | 0.0001 | 0.000106 |


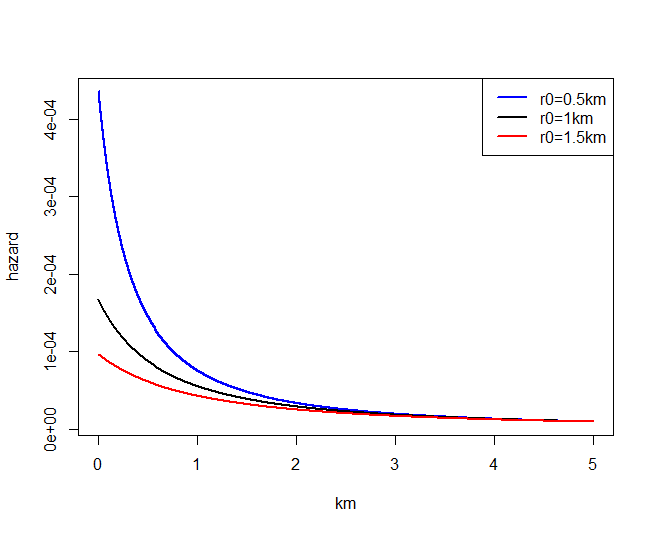


**Figure S2.6.** Visualization of the relationship between the infection hazard (y-axis) and distance (x-axis).

## S3. Detailed results: Response per participant

### ‘Validation’ of behaviour survey

Figure S3.1 presents the responses to three validation questions directed at survey participants regarding the different farmer profiles. The questions address three constructs of the TPB: (1) subjective norms, exploring whether the farmer’s management decisions are influenced by others’ opinions; (2) attitudes towards innovation, assessing the likelihood of adopting new farming technologies; and (3) perceived behaviour control, gauging the farmer’s confidence in their own farming abilities. These questions serve to verify if respondents had a consistent understanding of the farmer profiles.


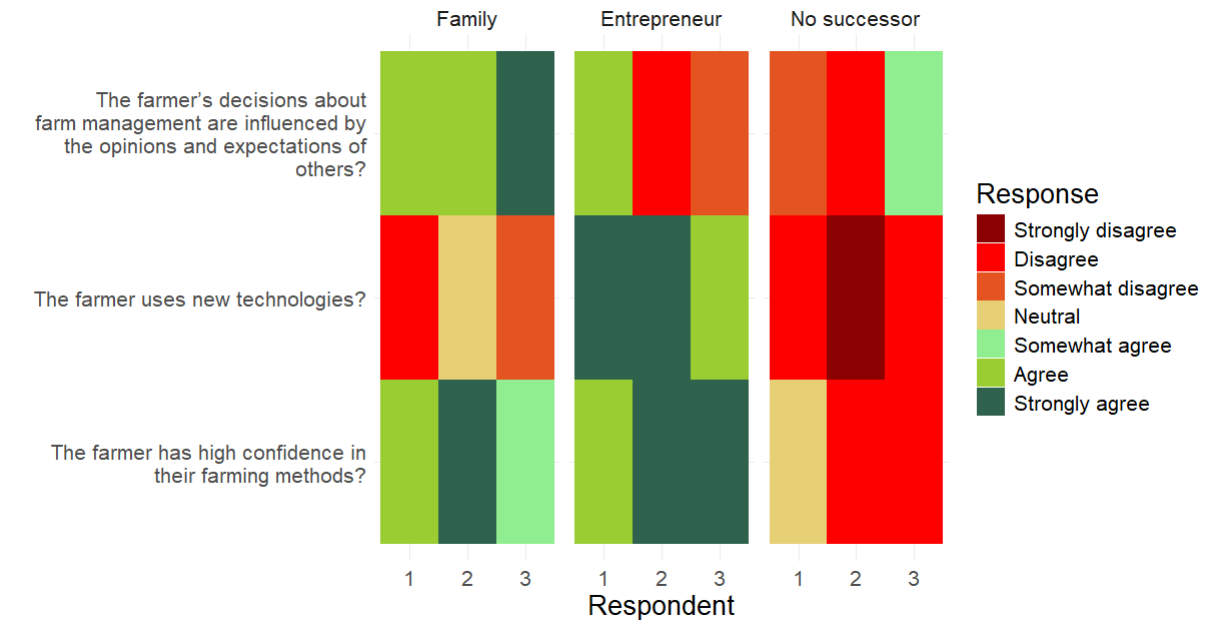


Figure S3.1: Survey respondent agreement on behavioural traits across farmer profiles.

The responses suggest that participants broadly shared similar perceptions of the profiles. For instance, all respondents agree that the “family” farmer’s decisions are influenced by external opinions (e.g., family, friends), that this farmer is unlikely to adopt new technology, and that they have confidence in their established methods. Across seven of the nine instances, responses fell consistently along a similar spectrum of agreement or disagreement, indicating an aligned understanding of the behavioural traits associated with the profiles.

However, divergence was observed in responses to the subjective norms question regarding the “entrepreneur” and “no successor” profiles. This inconsistency may stem from varying interpretations of “influence by others”. One hypothesis for these discrepancies could be that the concept of “influence by others” is interpreted differently depending on the respondent’s own perspective on entrepreneurial independence or social isolation in succession planning. The absence of precise guidance on what constitutes "influence by others" in the survey could have led respondents to rely on personal or cultural assumptions, adding to the variability in responses. The brevity of the profiles—kept short for survey efficiency—may also have contributed to these differences in interpretation.

For the "entrepreneur" profile, respondents may perceive entrepreneurs as inherently self-reliant. However, the profile description suggests a farmer influenced by financial stakeholders and a network of other successful farmers and agribusiness experts. This duality can create ambiguity, leading to conflicting views on the degree to which the entrepreneur farmer is influenced by others in their decision-making.

Similarly, the "no successor" profile may invite varied interpretations. The profile description suggests that the farmer is influenced by others who prioritise efficiency and financial outcomes, while paying less attention to those who advocate for sustainability or ethical practices. Some respondents might interpret a lack of succession planning as a sign of isolation or disengagement from community influences. Others, however, might interpret the lack of a successor as increasing a farmer's reliance on external guidance (e.g., from advisers, neighbours, or community leaders) for day-to-day decisions, thus aligning with agreement on subjective norms.

This highlights the importance of defining concepts like "influence by others" more precisely in future surveys to minimise interpretative ambiguity.

# References

1. Boender GJ, Hagenaars TJ. Common features in spatial livestock disease transmission parameters. Sci Rep. 2023;13(1):3550. Epub 20230302. <https://doi.org/10.1038/s41598-023-30230-w>.

2. Cador C, Rose N, Willem L, Andraud M. Maternally Derived Immunity Extends Swine Influenza A Virus Persistence within Farrow-to-Finish Pig Farms: Insights from a Stochastic Event-Driven Metapopulation Model. PLoS One. 2016;11(9):e0163672. Epub 20160923. <https://doi.org/10.1371/journal.pone.0163672>.

3. Pitzer VE, Aguas R, Riley S, Loeffen WL, Wood JL, Grenfell BT. High turnover drives prolonged persistence of influenza in managed pig herds. J R Soc Interface. 2016;13(119). <https://doi.org/10.1098/rsif.2016.0138>.

4. Widgren S, Bauer P, Eriksson R, Engblom S. SimInf: An R Package for Data-Driven Stochastic Disease Spread Simulations. Journal of Statistical Software. 2019;91(12):1 - 42. <https://doi.org/10.18637/jss.v091.i12>.

5. Olsen CW, Brown IH, Easterday BC, Van Reeth K. Swine influenza2006. 469-82 p.

6. Wagening University and Research. Agrimatie - informatie over de agrosector (Dutch) 2024 [10-10-2024]. Available from: <https://agrimatie.nl/SectorResultaat.aspx?subpubID=2232&sectorID=2255>.

7. Boender GJ, Hagenaars TJ, Bouma A, Nodelijk G, Elbers AR, de Jong MC, et al. Risk maps for the spread of highly pathogenic avian influenza in poultry. PLoS Comput Biol. 2007;3(4):e71. Epub 20070305. <https://doi.org/10.1371/journal.pcbi.0030071>.

8. Baudon E, Peyre M, Peiris M, Cowling BJ. Epidemiological features of influenza circulation in swine populations: A systematic review and meta-analysis. PLoS One. 2017;12(6):e0179044. Epub 20170607. <https://doi.org/10.1371/journal.pone.0179044>.

9. Toni T, Welch D, Strelkowa N, Ipsen A, Stumpf MP. Approximate Bayesian computation scheme for parameter inference and model selection in dynamical systems. J R Soc Interface. 2009;6(31):187-202. <https://doi.org/10.1098/rsif.2008.0172>.
